# FACTORS IMPACTING EMERGENCY PREPAREDNESS AMONG COMMUNITY-DWELLING OLDER ADULTS

**DOI:** 10.1093/geroni/igac059.2309

**Published:** 2022-12-20

**Authors:** Alisha Thompson, Kristina Little, Youn Kyoung (Lily) Kim

**Affiliations:** Louisiana State University, Baton Rouge, Louisiana, United States; Louisiana State University, Baton Rouge, Louisiana, United States; Louisiana State University, Baton Rouge, Louisiana, United States

## Abstract

Natural and manmade emergencies have become more frequent over the past 20 years and will continue to pose serious risks to public health and safety. Older adults are more adversely affected by—and less prepared for—emergencies than younger adults. However, little is known about the risk factors impeding emergency preparedness among older adults. This study uses the ecological systems approach to explore factors associated with emergency preparedness and how those factors influence adults 60 and older. The study analyzed cross-sectional data taken from 690 community-dwelling older adults who participated in Wave 5 of the National Poll on Healthy Aging. The sample was broken down into the following two groups for comparison: individuals aged 60–69 (n = 383) and individuals 70 or above (n = 307). The self-reported measures of sociodemographic characteristics, physical health, mental health, and previous experience of a disaster were utilized via regression analysis to predict emergency preparedness. Emergency preparedness was assessed using X dichotomous questions (60-69: M = X; 70+: M = X). The results revealed that living alone and having a Hispanic background were negatively associated with emergency preparedness among those aged 60–69, while mental health status was positively associated with emergency preparedness among those aged 70+. Previous experience of a disaster positively impacted emergency preparedness among the sample. Implications for policy and practice focus on shifting the perspective of the disproportional risks for older adults around emergencies to one that values and supports older adults’ strengths and insights.

